# Awareness of the Role of Physiotherapy in Treating Gynecological and Obstetric Issues Among Gynecologists: A Cross-Sectional Study

**DOI:** 10.3390/ijerph22111676

**Published:** 2025-11-04

**Authors:** Sami Elmahgoub, Adel El Taguri, Aseel Aburub, Mohamed I. Mabrouk, Márta Hock, Viktória Prémusz

**Affiliations:** 1 Physiotherapy Department, Faculty of Allied Medical Sciences, Applied Science Private University, Amman 11937, Jordan; a_abualrob@asu.edu.jo (A.A.); m_mabrouk@asu.edu.jo (M.I.M.); 2 Community Medicine Department, Faculty of Medicine, University of Tripoli, Tripoli 13932, Libya; tajoury@doctor.com; 3 Institute of Physiotherapy and Sports Science, Faculty of Health Sciences, University of Pécs, H-7621 Pécs, Hungary; hock.marta@etk.pte.hu; 4 Physical Activity Research Group, János Szentágothai Research Center, University of Pécs, H-7624 Pécs, Hungary; premusz.viktoria@pte.hu

**Keywords:** Health Knowledge, Attitudes, Practice, Gynecologists, Obstetricians, Physical Therapy Modalities, Referral and Consultation, Women’s Health, Libya

## Abstract

**Background:** Physiotherapy plays a crucial role in managing women’s health conditions, such as pelvic pain and postpartum recovery. However, its integration into standard care relies heavily on the awareness and referral practices of gynecologists and obstetricians, which may be suboptimal. **Objective:** To investigate the awareness, attitudes, and referral practices of gynecologists and obstetricians in Libya regarding the role of physiotherapy in women’s health. **Methods:** A cross-sectional study was conducted in public and private hospitals in Tripoli, Libya. A total of 100 practitioners were invited to participate. A self-administered questionnaire assessed demographics, awareness, attitudes, and practices. **Results:** The response rate was 67% (*n* = 67/100). The results revealed a key disparity: while the vast majority of respondents (94.1%) acknowledged the importance of physiotherapy in women’s health and 76.1% reported a willingness to refer patients, only 67.1% perceived physiotherapists as competent to manage these conditions. This indicates a significant gap in understanding specific physiotherapy techniques. Awareness percentage was significantly influenced by factors such as the type of hospital and prior exposure to physiotherapy education. **Conclusions:** A striking disparity was found between high general awareness (94.1%) and significantly lower perceived competence (67.1%) and knowledge of its role in specific conditions. This gap between general approval and specific understanding directly creates the critical barrier to referral. To improve interdisciplinary collaboration and patient access to care, enhanced educational initiatives for physicians, the development of clear clinical guidelines, and the establishment of dedicated women’s health physiotherapy clinics are strongly recommended.

## 1. Introduction

Physiotherapy in gynecology and obstetrics is a specialized field dedicated to promoting women’s health throughout all life stages, from adolescence to post-menopause [1]. The need for these services is underscored by the high global prevalence of conditions they address; for instance, pelvic floor dysfunction affects a substantial proportion of women worldwide, leading to significant quality-of-life issues [2]. Within Libya, data is limited, but the burden of postpartum complications and chronic pain is believed to be considerable, highlighting a major gap in accessible care. This area of practice addresses a wide range of conditions unique to women, including pelvic floor dysfunction, prenatal and postnatal care, and chronic pain management [2,3]. Women’s health physiotherapists possess extensive knowledge and expertise in managing issues related to pelvic health, pregnancy, and gynecological care [3,4]. According to leading international professional associations, the scope of practice for physiotherapists in this field includes the assessment and management of these conditions through specialized interventions such as pelvic floor muscle training, manual therapy, and therapeutic exercise [5]. In Libya, while the physiotherapy profession is established, formal sub-specialization in women’s health is not yet widespread, making awareness among referring physicians even more critical. During pregnancy, women undergo significant physiological and hormonal changes that can lead to musculoskeletal discomfort and other complications [6,7]. In this context, physiotherapy plays a crucial role in alleviating these challenges through various interventions. For instance, during prenatal care, physiotherapists assess and treat common conditions such as back pain and pelvic pain by providing specialized exercises and manual therapy aimed at reducing discomfort, improving posture, and enhancing pelvic floor function [7,8]. Additionally, techniques like breathing exercises and relaxation methods can be highly beneficial during labor [9]. After childbirth, physiotherapy supports the recovery of pelvic floor muscles that may have been affected, helping women regain strength, alleviate discomfort, and manage or prevent urinary incontinence [2,6]. Emphasizing the importance of physical activity, physiotherapy promotes safe and effective exercise to reduce the risk of gestational diabetes and pelvic pain while also enhancing cardiorespiratory endurance and mental well-being [10]. Beyond obstetric care, physiotherapy addresses various gynecological issues, offering non-invasive, evidence-based solutions that enhance overall physical well-being and improve quality of life [2,3]. For example, it effectively treats pelvic floor dysfunctions such as urinary incontinence and pelvic organ prolapse by strengthening pelvic floor muscles and restoring normal function [3,11]. Techniques like manual therapy and therapeutic exercises are also employed to manage pelvic pain [2], and physiotherapy is vital for rehabilitation following gynecological surgeries [12]. By delivering tailored interventions and exercises, women’s health physiotherapy not only optimizes function and alleviates pain but also significantly enhances the overall quality of life for women throughout their lives [2,4].

The awareness and understanding of gynecologists and obstetricians regarding the benefits and applications of physiotherapy in women’s health are critical for fostering effective collaboration across disciplines and ensuring that patients receive optimal care [1,2]. Effective interdisciplinary collaboration in healthcare is built upon a clear understanding of professional roles and teamwork. A professional role encompasses the specific skills, responsibilities, and scope of practice unique to a healthcare discipline. For collaboration to be successful, a mutual awareness and understanding of these roles between different professions is essential. Interdisciplinary collaboration can be defined as a cooperative process where different professionals work jointly toward shared patient goals, integrating their distinct knowledge. The literature indicates that such collaboration is increased by factors including positive inter-professional attitudes, the presence of formal referral pathways, and opportunities for joint education and communication [13]. Numerous studies have examined the level of awareness among these healthcare professionals, revealing a range of knowledge and integration of physiotherapy services within gynecological and obstetric practices [3,4]. Research indicates that many gynecologists and obstetricians recognize the important role physiotherapy can play in managing various obstetric and gynecological conditions [1,6]. For instance, a study conducted in Pakistan found that most obstetricians and gynecologists acknowledged the vital role of physiotherapy during pregnancy and postpartum [6]. Similarly, research in Ghana demonstrated that many doctors understand physiotherapy’s importance in obstetric care, particularly in the postnatal period [4]. However, despite this general awareness, gaps remain in understanding specific conditions and advanced physiotherapy techniques [14,15]. A study in Nigeria revealed that while many obstetricians and gynecologists possess a basic understanding of physiotherapy’s role, their familiarity with specific treatable conditions is often limited [15]. Another study in Pakistan indicated that although many practitioners are aware of fundamental interventions like Kegel exercises, fewer are knowledgeable about advanced techniques such as biofeedback and interferential therapy [14]. This disparity in awareness significantly impacts referral practices; studies show that even when awareness is high, referral rates often do not reflect this knowledge [12,14]. For example, although many gynecologists understand the role of physiotherapy in managing stress urinary incontinence, actual referral rates for these services frequently fall short of expectations [14]. Several factors contribute to this gap, including limited knowledge of specific treatments, insufficient interdisciplinary collaboration, and concerns about costs or time constraints [15,16]. Awareness levels among gynecologists and obstetricians can also be influenced by various elements, such as the type of hospital setting (private versus public), years of experience, and exposure to physiotherapy during medical education [14,16]. Studies indicate that gynecologists in private hospitals tend to demonstrate higher levels of awareness compared to those in public hospitals [14]. Additionally, those who have encountered physiotherapy as part of their training are generally more informed about its benefits [14]. To bridge the gap between awareness and practice, it is essential to enhance education and promote interdisciplinary collaboration [14,15]. Clinical meetings, seminars, and workshops can play a significant role in improving communication and understanding between physiotherapists and gynecologists/obstetricians [15]. Moreover, incorporating physiotherapy education into medical curricula can further elevate awareness among future healthcare practitioners [14].

Awareness of physiotherapy’s role in managing obstetric and gynecological conditions varies significantly across different regions. Studies indicate a relatively high level of awareness among gynecologists and obstetricians in Europe [15]. For instance, in the UK, physiotherapy is often integrated into prenatal and postnatal care, with regular referrals made for pelvic floor rehabilitation [3]. In North America, particularly in the USA and Canada, awareness levels are generally high, especially among practitioners in urban settings where interdisciplinary approaches are more common [3]. However, even as gynecologists and obstetricians increasingly recognize the benefits of physiotherapy for conditions like pelvic pain and incontinence, referral practices often lag behind this awareness [3]. In Asia, the situation is more complex, as awareness varies widely across countries. In Pakistan, for example, awareness is improving, but significant gaps remain in knowledge regarding specific therapeutic techniques [16,17]. Research indicates that while many gynecologists acknowledge the importance of physiotherapy, their understanding of advanced techniques remains limited, and referral practices often do not reflect these awareness levels [16,17]. In Africa, awareness levels tend to be lower compared to more developed regions, primarily due to limited access to physiotherapy services and a lack of structured training for medical professionals [3,12]. Studies conducted in Ghana reveal that although there is some awareness of physiotherapy’s role, the practical integration of these services into clinical practice is frequently obstructed by systemic barriers [3,12]. Overall, these regional disparities highlight the need for improved education and collaboration to enhance the integration of physiotherapy in women’s healthcare globally.

Despite the growing recognition of physiotherapy’s importance in women’s health, there is a significant lack of data on the awareness levels of gynecologists and obstetricians in Libya regarding its benefits. Current literature does not provide a clear understanding of how well these healthcare professionals grasp the role of physiotherapy in managing obstetric and gynecological conditions within the Libyan context. The primary objective of this cross-sectional study was to explore the awareness, attitudes, and referral practices of gynecologists and obstetricians in Tripoli, Libya, regarding the role of physiotherapy in women’s health. A secondary objective was to identify factors associated with their awareness and willingness to refer. We hypothesized that gynecologists and obstetricians in Tripoli, Libya, would have a favorable awareness of physiotherapy’s role in managing obstetric and gynecological conditions, yet demonstrate significant knowledge gaps regarding specific techniques and referral practices. This hypothesis is grounded in the need for enhanced interdisciplinary collaboration to improve patient outcomes.

## 2. Materials and Methods

### 2.1. Participants

The participants in this cross-sectional study comprised gynecologists and obstetricians working in both public and private hospitals in Tripoli, Libya. Given the focus on healthcare professionals, patient and public involvement was not applicable in this context. The insights from doctors were deemed sufficient to address the research questions posed. A convenience sampling strategy was employed. Inclusion criteria required participants to have a minimum of one year of current practice experience. Ethical approval for this study was obtained from the Institutional Review Board at the Faculty of Applied Medical Sciences, Applied Sciences Private University (AMS-2024-14), ensuring that the research adhered to ethical guidelines and standards for research involving human participants.

### 2.2. Procedures

The researchers invited a total of 100 doctors to participate in this study. Before consenting to participate, all participants received an information sheet outlining the study’s purpose and objectives, ensuring they were well-informed about their involvement. The researchers also spent time explaining and clarifying the questionnaire to each participant, addressing any questions or concerns they had. Consent was obtained from each individual before they began filling out the questionnaire, affirming their willingness to contribute to the research.

Participants typically required between 10 and 20 min to fill out the questionnaire. Unfortunately, 33 individuals were excluded from the study due to either declining participation or failing to complete the questionnaire in full. This careful process aimed to enhance the quality of the data collected while respecting the autonomy of each participant. To minimize interviewer bias, a standardized explanation script was used during all participant interactions.

### 2.3. Questionnaire

A self-administered questionnaire adapted from a standardized questionnaire used in previous work [3,15] was structured into two distinct sections: the first part included personal information and occupational details of the participants, such as age, sex, years of experience, workplace, clinical status, highest education level, and academic qualifications. The second part included 33 awareness questions divided into three subcategories: knowledge, attitude, and practice. The response format for these questions was a 4-point Likert scale (Strongly Agree, Agree, Disagree, Strongly Disagree). The knowledge section included self-reported perceptions aimed at gathering essential information, such as “Does physiotherapy have a role in obstetrics and gynecology?” and “Is your knowledge of the role of physiotherapy based on experience, studying, or both?”. The attitude section assessed doctors’ perception regarding physiotherapy, with statements such as, “Physiotherapy is time-demanding” and “Physiotherapists are not competent to manage my patients.” Finally, the practice section included questions about practical aspects, such as “Have you worked with a physiotherapist in management of obstetric patients?” and “Are you referring your cases to physiotherapy?” To ensure cross-cultural validity, the questionnaire underwent a rigorous translation and adaptation process following established guidelines [16]. The process involved independent translation by two bilingual translators, back-translation by two other blinded translators, and a final review by an expert committee to achieve semantic and conceptual equivalence. All participants were fluent in Arabic, the final language of administration.

### 2.4. Statistical Analysis

Data were analyzed using the Statistical Package for the Social Sciences (SPSS) version 23.0. Results for all collected information were presented as both numbers and percentages.

## 3. Result

### 3.1. Demographic Information

A total of 67 doctors working in public hospitals, private clinics, or both in Tripoli participated in this study. The majority of participants were female (85.1%) and under 45 years old (74.6%). All demographic details of participants are illustrated in Table 1.

### 3.2. Gynecologists and Obstetricians’ Awareness

Most of the participants (94.1%) have a good understanding of the importance of physiotherapy in treating gynecological and obstetric conditions. Furthermore, many doctors reported an increased awareness of the physiotherapy role. A significant percentage of doctors (53.7%) agreed that physiotherapy is a time-demanding profession, while 67.1% believed that physiotherapists are competent to treat their patients. Additionally, 76.1% of participants indicated that they would refer their patients to physiotherapy, and 68.7% reported having previously worked with a physiotherapist. Details about doctors’ awareness are shown in Table 2.

## 4. Discussion

This study assessed the awareness percentage of obstetricians and gynecologists in Tripoli, Libya, regarding the role of physiotherapy in managing obstetric and gynecological conditions. The results were encouraging: 94.1% of the doctors surveyed recognized that physiotherapy is important for helping patients with issues related to pregnancy, childbirth, and women’s health in general. Moreover, 76.1% expressed willingness to refer their patients to a physiotherapist, which highlights their recognition of physiotherapy as part of clinical practice. However, this high general awareness and willingness to refer masked a critical gap in specific knowledge. While 94.1% saw a general role for physiotherapy, only 67.1% perceived physiotherapists as competent to manage their patients, and awareness was strikingly low for specific gynecological conditions such as hysterectomy (22.4%), uterine prolapse (32.9%), and pelvic inflammatory disease (32.9%). This strong general understanding among these healthcare professionals about how physiotherapy can contribute positively to patient care, coupled with this clear knowledge gap, underscores that obstetricians and gynecologists are key players in integrating physiotherapy into patient care. The high percentage of awareness among participants (94.1%) suggests that these doctors understand the benefits of physiotherapy and reflects an evolving perspective on interdisciplinary collaboration, where physiotherapy is increasingly seen as an essential component of comprehensive women’s healthcare, not merely an adjunct [18,19]. This acknowledgment can lead to improvement of overall patient outcomes [17].

When we examine the findings of other studies worldwide, the high awareness percentage (94.1%) in Tripoli appears quite impressive. In places like the UK and other parts of Europe, physiotherapy is often part of the routine care for women before and after childbirth, so awareness is generally high [20]. In North America, especially in cities where doctors often work together across different fields, you see a similar level of understanding [21]. However, it is worth noting that even when doctors are aware of physiotherapy, they do not always refer patients as often as you might expect. On the other hand, in some Asian countries, the picture can be quite different. For instance, a study in Pakistan showed that while a high percentage of gynecologists were aware of physiotherapy, they did not always refer patients for treatment as often as they could have [14]. Another study pointed out that even when doctors recognize the importance of physiotherapy, they may not be familiar with the more advanced techniques [17]. Further, in Africa, awareness tends to be lower than in more developed regions, mainly because there are not as many physiotherapy services available and medical professionals do not always acquire specific training in this area, they found that while doctors were generally aware of and had positive attitudes toward physiotherapy, there was still a need for more education to make sure it is used effectively in women’s health [18]. Furthermore, the findings from Tripoli offer a promising model for other developing regions. The high baseline of general awareness and positive attitude toward collaboration, which exceeds levels reported in some studies across Asia and Africa [3,12,14,18], provides a unique and advantageous starting point. This suggests that in similar contexts, the primary challenge may not be initiating interdisciplinary dialog from scratch, but rather building upon an existing foundation of acceptance. The strategic focus, therefore, can shift directly to addressing the specific knowledge gaps we identified. The Libyan experience demonstrates that with a foundation of positive physician attitudes, targeted educational interventions, and the development of clear clinical pathways, it can be the most efficient strategy for integrating women’s health physiotherapy into standard care.

This study aligns with findings from Europe and North America, indicating a general recognition of physiotherapy’s role in women’s health. But compared to parts of Asia and Africa, the awareness percentage is higher. This gap between awareness and practice is not unique to Libya; it mirrors findings from other regions, where studies have also documented high awareness that does not translate into consistent referral rates [3,14,15]. It is important to remember that comparing studies like this can be tricky due to variations in study methodologies, sample characteristics, and healthcare systems. Nevertheless, the Libyan data suggest a promising foundation for further integrating physiotherapy into obstetric and gynecological care in Libya. So, Further studies are needed to explore referral practices and barriers to collaboration in the Libyan context.

Given that the vast majority of doctors in this study acknowledged the importance of physiotherapy (94.1) and most reported willingness to refer (76.1), we have a real opportunity to make it easier for them to connect their patients with physiotherapists. This could mean creating simple referral forms, setting up clear guidelines, and encouraging doctors and physiotherapists to talk directly to each other. Our findings also support the idea of having physiotherapists working right in the same clinics or hospitals as the obstetricians and gynecologists. This would make it easier for everyone to work together, improve patient access to physiotherapy, and really focus on treating the whole person. Even though awareness is high, there might still be some gaps in what doctors know about specific physiotherapy techniques, as our results revealed that a limited number of participants’ doctors were knowledgeable about many gynecological conditions and postnatal care. Continued awareness campaigns, including workshops, seminars, and meetings, are essential to further educate obstetricians and gynecologists about the benefits of physiotherapy in women’s health so they can better understand each other’s roles and expertise. To make all of this work, we also need to make sure there are enough resources, like qualified physiotherapists, the right equipment, and stable funding for physiotherapy services.

Based on this study’s findings, there are several recommendations to better integrate physiotherapy into women’s healthcare in Libya. First off, develop clear, evidence-based guidelines for common obstetric and gynecological conditions, which explicitly include indications for physiotherapy referral and intervention. These guidelines should be created through a collaborative, interprofessional effort. Secondly, design and implement interprofessional education programs, such as workshops and seminars, that move beyond general awareness and instead focus on specific physiotherapy techniques and their measurable outcomes for under-recognized conditions, such as rehabilitation following hysterectomy and conservative management of pelvic organ prolapse. Thirdly, establish dedicated physiotherapy clinics within or near obstetric and gynecological care facilities to improve patient access and facilitate collaboration between providers. Fourthly, address the shortage of qualified physiotherapists by increasing the number of specialized training programs and creating incentives to attract professionals to this field. Fifthly, raising public awareness through campaigns that educate women about the role and importance of physiotherapy in treating various gynecological and obstetric conditions. Sixthly, identify and address systemic and cultural barriers that hinder people from receiving physiotherapy, like cost, transportation issues, or cultural beliefs. Seventhly, regular check-ups or audits on the impact of the above-mentioned initiatives on patient outcomes and healthcare costs. Eighthly, advocate for the government and policymakers to issue policies that support physiotherapy, like including it in health insurance plans and setting national standards for how it is practiced. Finally, promote further research to explore the efficacy and implementation of physiotherapy for obstetric and gynecological conditions within the Libyan context.

However, this study has limitations. The use of a convenience sampling method, confined solely to the urban center of Tripoli, introduces potential selection bias and significantly limits the generalizability of the findings. Consequently, the results may not be representative of all regions of Libya, which may have different healthcare resources and practitioner profiles, nor can they be generalized to other countries with differing healthcare systems. The questionnaire design may not have comprehensively addressed all aspects of physiotherapy integration, and the reliance on self-reported data introduces the possibility of response bias. The questionnaire format may not allow for in-depth exploration of attitudes and beliefs regarding physiotherapy, and the cross-sectional design limits the ability to establish causal relationships. Finally, the study was conducted solely in Tripoli, which may not represent all regions in Libya. Furthermore, while the questionnaire underwent a thorough translation and adaptation process, future studies could benefit from adhering to a full, internationally recognized cross-cultural validation guideline, such as those proposed by Beaton et al. [16].

Future research should be strategically directed along two key avenues. First, there is a compelling need for international comparative studies that benchmark awareness and referral practices in Libya against those in other countries, particularly within North Africa and the Middle East. Such research would provide critical insights into how cultural norms, medical education curricula, and healthcare system structures influence the integration of physiotherapy services. Second, to move from assessing awareness to demonstrating efficacy, longitudinal studies are essential. These studies should track whether targeted educational interventions for physicians on specific physiotherapy techniques directly lead to sustained improvements in referral rates and, most importantly, to enhanced patient outcomes for conditions like urinary incontinence and pelvic pain.

## 5. Conclusions

This study assessed the awareness of obstetricians and gynecologists in Tripoli, Libya, regarding the role of physiotherapy in managing obstetric and gynecological conditions. The high awareness of the gynecologists and obstetricians and their willingness to refer patients indicates a positive trend toward interdisciplinary collaboration in women’s healthcare.

However, gaps in knowledge about specific physiotherapy techniques remain, highlighting the need for ongoing education and awareness campaigns. Recommendations include establishing clear guidelines, creating dedicated physiotherapy clinics, and addressing barriers to access, such as cost and transportation. Future research should focus on exploring awareness levels and referral practices further, as well as the impact of physiotherapy on patient outcomes. These efforts aim to foster a more integrated approach to women’s health, ultimately improving care quality and health outcomes for women in Libya.

## Figures and Tables

**Table 1:** Participants’ demographic characteristics.

Variables		*n*	%
Age	25–35	25	37.3
	35–45	25	37.3
	>45	17	25.4
Sex	M	9	13.4
	F	57	85.1
Years of experience	0–5	15	22.4
	5–10	13	19.4
	10–15	16	23.9
	15–20	7	10.4
Work place	Public hospital	14	20.9
	Private hospital	12	17.9
	Both	41	61.2
Clinical status	SHO	25	37.3
	Registrar	12	17.9
	Specialist	20	29.9
	Consultant	10	14.9
Highest education level			
A- Academic	Postgraduate diploma	14	20.9
	Masters	9	13.4
	PhD	14	20.9
B- Clinical	Libyan board	12	17.9
	Arab board	25	37.3
	Other board	9	13.4

**Table 2:** Participants’ awareness of physiotherapy practice.

Variables	Agree	Disagree
	*n* (%)	*n* (%)
Does physiotherapy have a role in obstetrics and gynecology?	63 (94)	4 (6)
The role of physiotherapy was based on?		
Experience	25 (37.4)	42 (62.6)
Studying	9 (13.4)	58 (86.6)
Both	32 (47.2)	35 (52.2)
There are enough physiotherapists in my hospital/clinic to cover the obstetrics and gynecology ward	32 (47.8)	35 (52.3)
Presence of a physiotherapy clinic within the vicinity of the hospital of practice	35 (52.2)	32 (47.8)
The physiotherapy service is too expensive	58 (86.6)	9 (13.4)
Based on your knowledge, does physiotherapy have a role in the management of the following conditions: A- Gynecologic conditions:		
Uterine prolapse	22 (32.9)	45 (67.1)
Pelvic inflammatory disease	22 (32.9)	45 (67.1)
Hysterectomy	15 (22.4)	52 (77.6)
Cervical incompetence	60 (89.6)	7 (10.4)
B- Obstetric practice:		
Antenatal care	46 (68.7)	21 (31.3)
Parturition	65 (97)	2 (3.0)
Postnatal care	35 (52.2)	32 (47.8)
Have a physiotherapist as a close friend	46 (68.7)	21 (31.3)
A physiotherapist should go on a ward round with doctors.	40 (59.7)	27 (40.3)
The physiotherapy training/clinical department is available in my hospital	35 (52.2)	32 (47.8)
The following are types of postnatal exercises:		
Breathing exercises	59 (88)	8 (12)
Kegel’s exercises	63 (94)	4 (6.0)
Pelvic floor exercises	63 (94)	4 (6.0)
Abdominal exercises	57 (85)	10 (15)
Physiotherapy is time-demanding	36 (53.7)	31 (46.3)
Physiotherapists are not competent to manage my patient	22 (32.9)	45 (67.1)
How is the awareness of obstetricians and gynecologists raised about the role of physiotherapy in obstetrics and gynecology?		
Awareness campaigns	57 (85)	10 (15)
Awareness lectures	62 (92.5)	5 (7.5)
Doctors’ meetings with physiotherapists	58 (86.6)	9 (13.4)
Awareness leaflets	54 (80.6)	13 (19.4)
Physiotherapists have been adequate in their inter-professional relationships	44 (65.7)	23 (34.3)
Physiotherapists should be allowed to attend some surgical operations for gynecologic and obstetric patients	31 (46.3)	36 (53.7)
Physiotherapy may not contribute significantly to complete well-being with drugs and instructions	27 (40.3)	40 (59.7)
Worked with a physiotherapist in the management of obstetric patients	46 (68.7)	21 (31.3)
Are you referring your cases to physiotherapy?	51 (76.1)	16 (23.9)
Physiotherapy has worsened the condition of my patients	16 (23.8)	51 (76.2)

## Data Availability

The original contributions presented in this study are included in the article. Further inquiries can be directed to the corresponding author.
